# Erratum to “Prevalence of Asthma and Chronic Obstructive Pulmonary Disease in Aboriginal and Non-Aboriginal Populations: A Systematic Review and Meta-Analysis of Epidemiological Studies”

**DOI:** 10.1155/2017/8419686

**Published:** 2017-11-05

**Authors:** Maria B. Ospina, Donald C. Voaklander, Michael K. Stickland, Malcolm King, Ambikaipakan Senthilselvan, Brian H. Rowe

**Affiliations:** ^1^School of Public Health, University of Alberta, Edmonton, AB, Canada; ^2^Division of Pulmonary Medicine, University of Alberta, Edmonton, AB, Canada; ^3^Department of Emergency Medicine, Faculty of Medicine & Dentistry, University of Alberta, Edmonton, AB, Canada

In the article titled “Prevalence of Asthma and Chronic Obstructive Pulmonary Disease in Aboriginal and Non-Aboriginal Populations: A Systematic Review and Meta-Analysis of Epidemiological Studies” [1], there were errors in the figures, where Figure 2 was missing from the article and replaced by Figure 5, and Figures 3 and 4 were reversed. The correct figures are shown below:

## Figures and Tables

**Figure 2:**
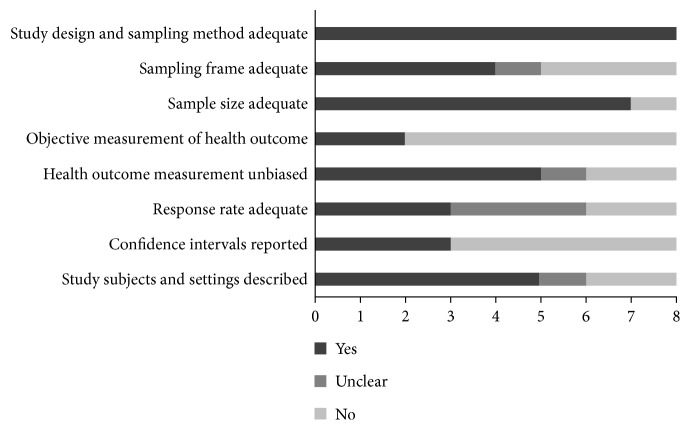
Summary of methodological characteristics of included studies.

**Figure 3:**
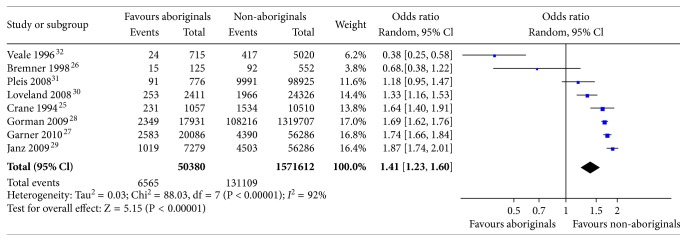
Meta-analysis of the comparison of asthma prevalence between Aboriginals and non-Aboriginals.

**Figure 4:**
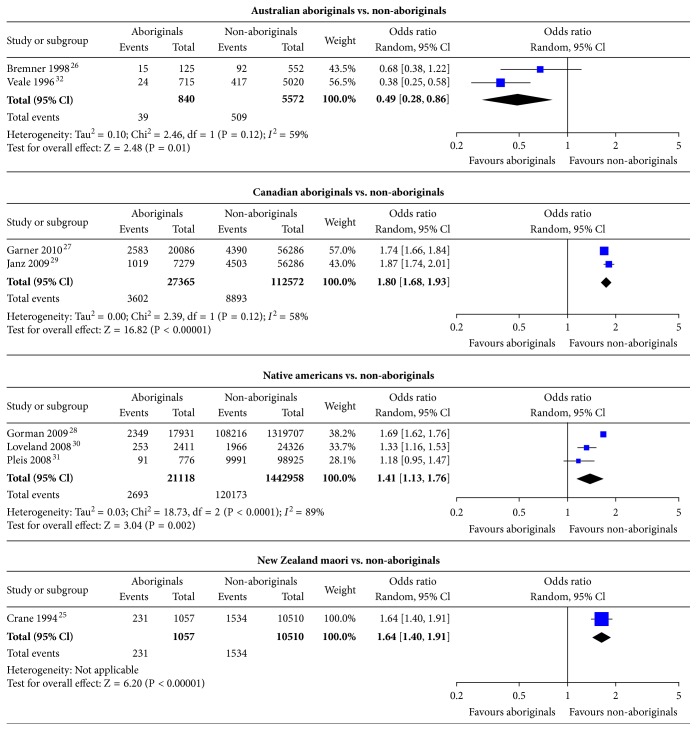
Subgroup analysis of the comparison of overall asthma prevalence between Aboriginals and non-Aboriginals by Aboriginal type.

## References

[1] Ospina M. B., Voaklander D. C., Stickland M. K., King M., Senthilselvan A., Rowe B. H. (2012). Prevalence of asthma and chronic obstructive pulmonary disease in Aboriginal and non-Aboriginal populations: a systematic review and meta-analysis of epidemiological studies. *Canadian Respiratory Journal*.

